# The soluble mannose receptor (sMR/sCD206) in critically ill patients with invasive fungal infections, bacterial infections or non-infectious inflammation: a secondary analysis of the EPaNIC RCT

**DOI:** 10.1186/s13054-019-2549-8

**Published:** 2019-08-02

**Authors:** Greet De Vlieger, Ilse Vanhorebeek, Pieter J. Wouters, Inge Derese, Michael P. Casaer, Yves Debaveye, Greet Hermans, Philippe Meersseman, Holger J. Møller, Greet Van den Berghe, Catherine Ingels

**Affiliations:** 1 Clinical Division of Intensive Care Medicine, UZ Leuven, Herestraat 49, 3000 Leuven, Belgium; 2 Laboratory of Intensive Care Medicine, Department of Cellular and Molecular Medicine, KU Leuven, Herestraat 49, 3000 Leuven, Belgium; 3 Department of General Internal Medicine, UZ Leuven, Herestraat 49, 3000 Leuven, Belgium; 4 Department of Clinical Biochemistry, Aarhus University Hospital, Aarhus, Denmark

**Keywords:** Invasive fungal infection, Inflammation, Soluble mannose receptor, sMR, Soluble CD206, sCD206, Macrophage activation

## Abstract

**Background:**

Invasive fungal infections (IFI) are difficult to diagnose, especially in critically ill patients. As the mannose receptor (MR) is shed from macrophage cell surfaces after exposure to fungi, we investigate whether its soluble serum form (sMR) can serve as a biomarker of IFI.

**Methods:**

This is a secondary analysis of the multicentre randomised controlled trial (EPaNIC, *n* = 4640) that investigated the impact of initiating supplemental parenteral nutrition (PN) early during critical illness (Early-PN) as compared to withholding it in the first week of intensive care (Late-PN). Serum sMR concentrations were measured in three matched patient groups (proven/probable IFI, *n* = 82; bacterial infection, *n* = 80; non-infectious inflammation, *n* = 77) on the day of antimicrobial initiation or matched intensive care unit day and the five preceding days, as well as in matched healthy controls (*n* = 59). Independent determinants of sMR concentration were identified via multivariable linear regression. Serum sMR time profiles were analysed with repeated-measures ANOVA. Predictive properties were assessed via area under the receiver operating curve (aROC).

**Results:**

Serum sMR was higher in IFI patients than in all other groups (all *p* < 0.02), aROC to differentiate IFI from no IFI being 0.65 (*p* < 0.001). The ability of serum sMR to discriminate infectious from non-infectious inflammation was better with an aROC of 0.68 (*p* < 0.001). The sMR concentrations were already elevated up to 5 days before antimicrobial initiation and remained stable over time. Multivariable linear regression analysis showed that an infection or an IFI, higher severity of illness and sepsis upon admission were associated with higher sMR levels; urgent admission and Late-PN were independently associated with lower sMR concentrations.

**Conclusion:**

Serum sMR concentrations were higher in critically ill patients with IFI than in those with a bacterial infection or with non-infectious inflammation. However, test properties were insufficient for diagnostic purposes.

**Electronic supplementary material:**

The online version of this article (10.1186/s13054-019-2549-8) contains supplementary material, which is available to authorized users.

## Background

Critical care medicine has evolved dramatically over the last decades, reducing mortality of patients with life-threatening diseases. Nevertheless, long-term outcomes remain poor and patients surviving the acute phase of critical illness are at risk for several complications, among which severe nosocomial infections [1]. In particular, invasive fungal infections (IFI) have emerged in this population and have been associated with an increased morbidity and mortality [1, 2]. Timely initiation of appropriate antifungal treatment is considered an important prognostic factor [3]. However, classical diagnostic tools such as culture and biopsy are invasive and time-consuming and show low sensitivity [4]. Biomarkers, such as serum fungal cell wall components, can be detected rapidly but have a variable sensitivity and a low specificity, especially in intensive care unit (ICU) patients [4, 5]. Moreover, empirical treatment in high-risk patients does not seem to improve clinical outcome [6, 7]. Hence, there is an increasing need for novel and accurate diagnostic tools for IFI during critical illness.

The innate immune system recognises fungal species by pathogen recognition receptors, such as dectin-1 and the mannose receptor (MR or CD206) [8]. The MR is an endocytic receptor present in macrophages, dendritic cells, and endothelial cells and is transported to the cell surface during inflammation. It contains several extracellular domains, each having their own binding capacities to sulfated glycans, carbohydrates, collagens, allergens, and pathogens [9]. Binding of a ligand to the receptor probably stimulates metalloproteinases to cleave the cell-bound receptor into a soluble form, which is shed into the circulation as soluble MR (sMR) [8]. This process is, among others, driven by dectin-1, which is activated by exposure to beta-d-glucan from the fungal cell wall [10]. The sMR shedding is documented in vitro in macrophages exposed to several fungal pathogens such as *Candida albicans*, *Aspergillus fumigatus* and *Pneumocystis carinii* [10, 11]. Elevated serum sMR has also been documented in patients with liver disease, sepsis, invasive pneumococcal disease and malignancies [12–18].

As MR expression and shedding increase when macrophages are exposed to fungi, we hypothesised that serum sMR concentration could be a valuable tool to diagnose IFI early in critical illness.

## Methods

### Study subjects

This is a secondary analysis from a multicentre randomised controlled trial (*n* = 4640) investigating the impact of accepting a macronutrient deficit during the first week of critical illness (EPaNIC; ClinicalTrials.gov NCT00512122) [19]. Patients were randomised to either receive parenteral nutrition (PN) to supplement insufficient enteral nutrition within 48 h after ICU admission (Early-PN, *n* = 2312) or to withhold supplemental PN in the first week of intensive care (Late-PN, *n* = 2328). The trial was conducted in seven adult ICUs from August 1, 2007, until November 8, 2010. The ethics committee of all participating hospitals (ML4190) and the Belgian authorities (EudraCT2007e000169e40) approved the study protocol and consent forms that included approval for additional secondary analyses. All patients or their designated representatives provided a written informed consent.

The trial showed improved outcomes with Late-PN as reflected by shorter duration of stay in ICU and hospital, fewer ICU-acquired infections, shorter duration of mechanical ventilation and renal replacement therapy and reduced healthcare-related costs [19, 20]. Furthermore, fewer Late-PN patients acquired an IFI as compared with Early-PN patients [21].

Of the 97 EPaNIC patients with a proven/probable IFI as defined by the European Organisation for Research and Treatment of Cancer/Mycosis Study Group (EORTC/MSG) criteria [22] or with invasive yeast infections as defined by the European Society of Clinical Microbiology and Infectious Diseases (ESCMID) guidelines [23], we excluded those patients who received empirically initiated antifungal treatment before microbiological confirmation of IFI, as well as non-survivors for whom autopsy could not confirm IFI diagnosis. Accordingly, 84 patients with proven/probable IFI remained eligible. These patients were matched for baseline characteristics with a group of patients with an ICU-acquired bacterial infection and a group of patients suffering from non-infectious inflammation, as well as with a group of healthy controls. In the patients with an ICU-acquired bacterial infection, an IFI was excluded by reviewing the patient files until 48 h after ICU discharge [21]. Non-infectious inflammation refers to the inflammation secondary to critical illness and tissue damage. In this group, patients did not acquire a new infection during their ICU stay.

**Fig. 1:**
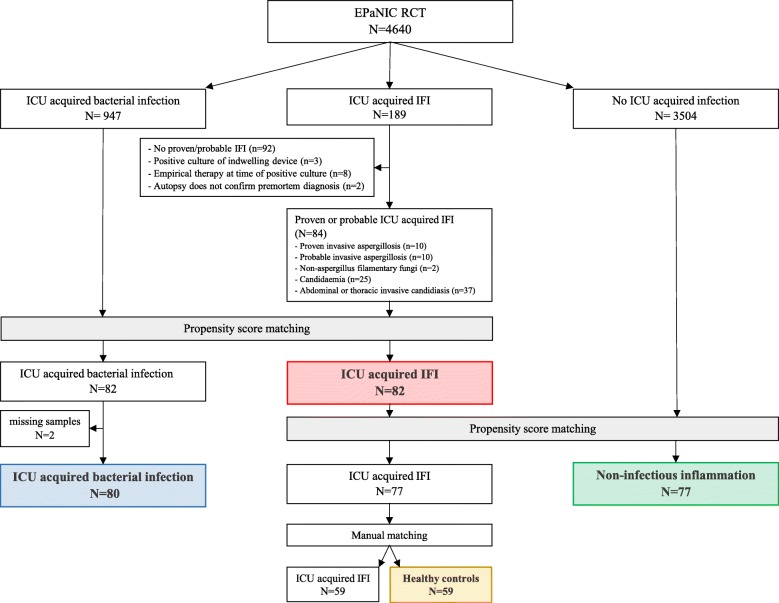
Consort diagram. ICU: intensive care unit, IFI: invasive fungal infection. Coloured boxes: by propensity score-matched patients and manually matched healthy controls

In a first step, patients with an IFI were matched to those with a bacterial infection by propensity score with a calliper of 0.1 and using EPaNIC-randomisation, acute physiology and chronic health evaluation II (APACHE-II) score [24], administration of steroids upon admission, sepsis at admission, age, admission diagnostic category (cardiac surgery, complicated surgery, trauma/burns, medical disease), emergency admission, body mass index (BMI), history of cirrhosis (Child-Pugh B/C), diabetes mellitus or malignancy and mechanical ventilation upon admission as covariates, in addition to the day of initiation of antimicrobial treatment for an ICU-acquired infection (which is antifungal treatment in the IFI group and antibiotic treatment in the patients with a bacterial infection). Of the 84 IFI patients, 82 patients could be propensity score matched to 82 patients who acquired a bacterial infection on a similar ICU day (Fig. 1). However, two patients with bacterial infection were eventually excluded due to unavailability of serum samples on the day that antibiotic treatment was initiated. In a second propensity score matching, 77 of the 82 retained IFI patients could similarly be matched with 77 patients with non-infectious inflammation with an ICU stay comparable to the day of initiation of antimicrobial treatment in the IFI group, based on a calliper of 0.1 and using the same covariates (Fig. 1). Finally, 59 of the IFI patients were matched with 59 healthy controls for age, BMI, history of malignancy, and gender (Fig. 1). Baseline characteristics were similar for all groups (Table 1).

**Table 1:** Baseline characteristics of the propensity score-matched patients and healthy controls

	Fungal infection (*n* = 82)	Bacterial infection (*n* = 80)	No infection (*n* = 77)	Healthy controls (*n* = 59)	*p* value
Age, median (IQR)	65 (54–75)	62 (48–72)	62 (52–73)	68 (60–75)	0.11
Gender, male (*n*, %)	58 (70.7%)	47 (58.8%)	47 (61.0%)	36 (61.0%)	0.40
BMI, median (IQR)	25.1 (22.7–28.4)	26.2 (23.1–29.4)	24.9 (22.5–28.4)	25.7 (23.0–28.0)	0.43
Malignancy (*n*, %)	25 (30.5%)	19 (23.8%)	19 (24.7%)	21 (35.6%)	0.39
Cirrhosis CP B/C (*n*, %)	10 (12.2%)	11 (13.8%)	8 (10.4%)		0.81
Diabetes mellitus (*n*, %)	12 (14.6%)	16 (20.0%)	8 (10.4%)		0.24
APACHE-II, median (IQR)	38 (31–42)	37 (31–40)	35 (31–40)		0.24
Sepsis at adm (*n*, %)	67 (81.7%)	65 (81.3%)	64 (83.1%)		0.95
Urgent admission (*n*, %)	77 (93.9%)	78 (97.5%)	73 (94.8%)		0.53
Diagnostic group (*n*, %)					0.09
Cardiac surgery	5 (6.1%)	7 (8.8%)	3 (3.9%)		
Complicated surgery	47 (57.3%)	43 (53.8%)	41 (53.3%)		
Trauma/burns	4 (4.9%)	10 (12.5%)	2 (2.6%)		
Medical disease	26 (31.7%)	20 (25.0%)	31 (40.3%)		
Steroids at adm (*n*, %)	28 (34.2%)	27 (33.8%)	27 (35.1%)		0.98
MV at adm (*n*, %)	69 (84.2%)	68 (85.0%)	61 (79.2%)		0.59
Randomisation Late-PN (*n*, %)	37 (45.1%)	39 (48.8%)	42 (54.6%)		0.49
Matching day, median (IQR)	6 (3–14)	8 (5–13)	7 (4–11)		0.10

Matching day refers to the day of antimicrobial initiation for patients with a new infection and matched ICU day for patients without a new infection. Statistical analyses have been performed with the Kruskal-Wallis test for continuous variables and with the chi-square test for categorical variables

*IQR* interquartile range, *BMI* body mass index, *CP* Child-Pugh, *APACHE-II* acute physiology and chronic health evaluation II, *adm* admission, *MV* mechanical ventilation, *PN* parenteral nutrition

### Serum sMR concentration

Daily morning serum samples had been collected during the EPaNIC-study, stored at − 80 °C until analysis. The sMR concentration was quantified with an in-house developed enzyme-linked immunosorbent assay (ELISA). We used a polyclonal anti-human MR antibody (R&D Systems, catalogue number AF2534, Minneapolis, MN, USA) as catching antibody and a biotinylated monoclonal anti-MR antibody (Acris Antibodies, Clone 7–450, catalogue number AM05589PU-S, Hereford, Germany) as the detection antibody. The detection method was described previously [18]. Previous reports show stable sMR concentrations when samples were stored at minus 80 °C for up to 9 months and for freeze-thaw cycles up till 7 times [18]. All laboratory staff was blinded for group allocation and other clinical data.

For patients, serum sMR concentrations were quantified on the day of initiating antimicrobial treatment (IFI or bacterial infection group) or matched ICU day (non-infectious inflammation group) and daily up to 5 days prior to this day, depending on sample availability. For healthy controls, one serum sample was analysed.

### Statistical analyses

Categorical data are presented as numbers and percentages. Numerical data are presented as medians and interquartile range (IQR) or as means and standard deviation (SD) as appropriate for distribution. The serum sMR concentrations were transformed by double square root to obtain a normal distribution and differences among groups were analysed with the use of Student *t* test, analysis of variance (ANOVA) and repeated-measures ANOVA. To identify determinants of the serum sMR concentrations, we performed a multivariable linear regression analysis using EPaNIC-randomisation, APACHE-II score, administration of steroids upon admission, sepsis at admission, age, admission diagnostic category (cardiac surgery, complicated surgery, trauma/burns, medical disease), emergency admission, BMI, history of cirrhosis (Child-Pugh B/C), diabetes mellitus or malignancy and mechanical ventilation upon admission as variables. Predictive properties of sMR were assessed via area under the receiver operating curve (aROC). Two-sided *p* values < 0.05 were considered significant.

Propensity score matching was performed with SPSS R-menu R3.1 (Foundation for Statistical Computing) in IBM SPSS Statistics 24.0.0.0 (SPSS, Chicago, IL)). Other statistical analyses were performed with JMP-software, version 14.0.0. (SAS-Institute Inc., Cary, NC).

## Results

### Serum sMR concentrations in critically ill patients and healthy controls

**Fig. 2:**
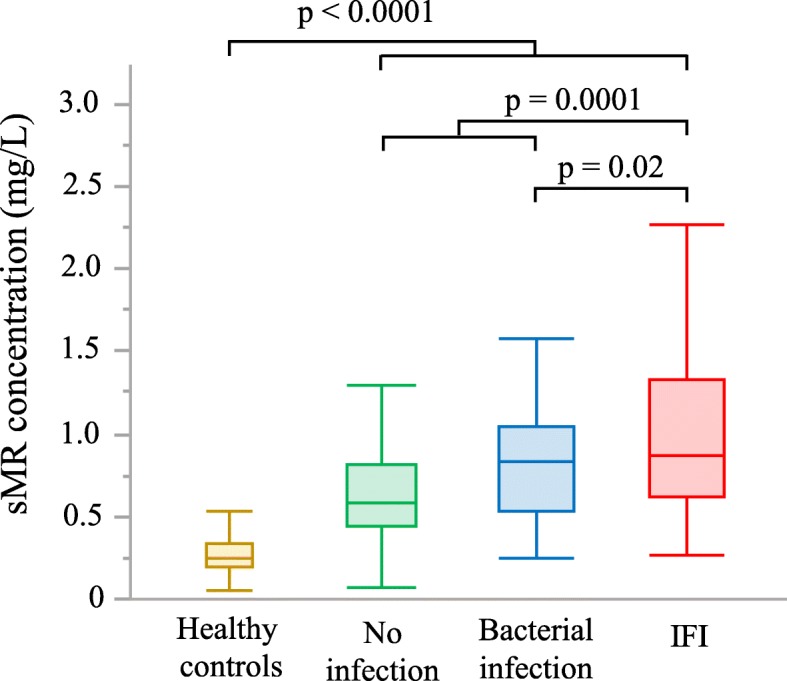
The sMR concentration on the day of antimicrobial initiation or matched ICU day. Serum concentrations are shown for healthy controls and for patients. For patients, concentrations are shown on the day of initiation of antimicrobial treatment in the groups with fungal or bacterial infection and on the matched day of ICU stay in the group with non-infectious inflammation. Statistical analyses have been performed with the student t-test after double square root transformation to obtain a normal distribution

On the day of antimicrobial initiation or matched ICU day, serum sMR concentrations were significantly different (all *p* ≤ 0.02) among the four groups, with a median (IQR) of 0.24 (0.20–0.33) mg/L in healthy controls, 0.58 (0.45–0.81) mg/L in patients with non-infectious inflammation, 0.84 (0.53–1.04) mg/L in patients with a bacterial infection and 0.87 (0.62–1.33) mg/L in patients with IFI (Fig. 2). The highest serum sMR concentrations were observed for patients with proven invasive aspergillosis (1.20 (0.57–1.51) mg/L) and for patients with candidaemia (1.14 (0.67–1.81) mg/L) (Table 2). The sMR serum concentrations for different sites of bacterial infections are available in the supplemental information (Additional file 1).

**Table 2:** Overview of the sMR concentrations in the different groups of patients with invasive fungal infections

	Number of patients	sMR (mg/L) (median and IQR)
Proven invasive aspergillosis	9	1.20 (0.57–1.51)
Probable invasive aspergillosis	10	0.78 (0.66–1.06)
Other filamentary fungi	2	0.82 (0.63–1.01)
Candida blood stream infection	24	1.14 (0.67–1.81)
Invasive candidiasis (abdominal, pleural or mediastinal)	37	0.84 (0.59–1.08)

*sMR* soluble mannose receptor, *IQR* interquartile range

**Fig. 3:**
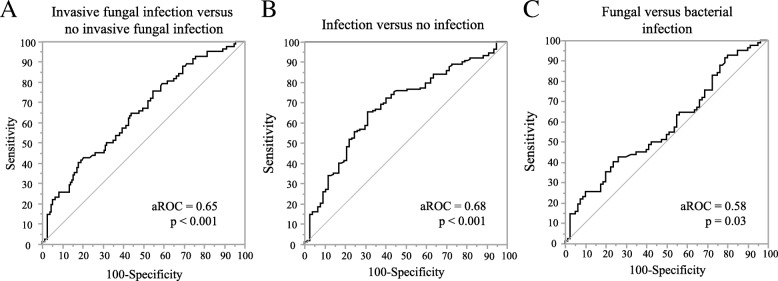
Receiver operating characteristic curves of sMR concentration to predict an invasive fungal or bacterial infection. **a**. aROC for sMR to differentiate invasive fungal infection from no invasive fungal infection. The optimal cut-off for sMR is 1.04 mg/L with a corresponding sensitivity of 40.2% and specificity of 82.2%. **b**. aROC for sMR to differentiate infection from no infection. The optimal cut-off for sMR is 0.71 mg/L with a corresponding sensitivity of 64.4% and specificity of 68.8%. **c**. aROC for sMR to differentiate an invasive fungal infection from a bacterial infection. The optimal cut-off is 1.04 mg/L with a corresponding sensitivity of 40.3% and specificity of 76.3%. aROC: area under the receiver-operating-curve

The aROC for predicting the presence of an IFI by serum sMR concentrations on the day of antimicrobial initiation or matched ICU day was 0.65 (*p* < 0.001) (Fig. 3). For predicting an infection (bacterial infection or IFI), the aROC was 0.68 (*p* < 0.001) (Fig. 3). When only taking patients with an infection into account, the aROC to differentiate an IFI from a bacterial infection was 0.58 (*p* = 0.03) (Fig. 3).

### Evolution of serum sMR concentrations over time

**Fig. 4:**
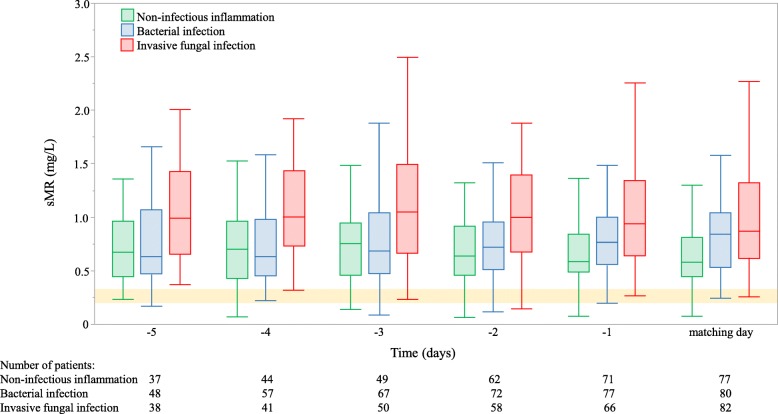
Evolution in serum sMR concentrations from 5 days before until matching day. Repeated measures ANOVA were done with double square root transformed data. *P*-values showed no difference in the evolution of sMR concentration in the three studied groups when assessing from 5 (*p* = 0.89), 4 (*p* = 0.44), 3 (*p* = 0.15) or 2 (*p* = 0.37) days before matching day until the matching day. Matching day refers to the day of antimicrobial initiation for patients with a new infection and matched ICU day for patients without a new infection. The horizontal yellow-shaded area represents the interquartile range of the sMR concentration in 59 matched healthy controls

In IFI-patients, serum sMR concentrations were already elevated as compared to other patient groups from 5 days prior to the day of antimicrobial initiation or matched ICU day onwards and remained stable over time (Fig. 4). For all five preceding days, serum sMR concentrations were significantly higher in patients with IFI as compared to patients without IFI and as compared to patients with a bacterial infection (Additional file 2). The predictive properties of the serum sMR concentration for identification of patients who acquired an IFI were comparable for the day of antimicrobial initiation or matched ICU day and all 5 days preceding this day (Additional file 3).

### Independent determinants of serum sMR concentrations in critically ill patients

In multivariable linear regression analysis, patient group (IFI, bacterial infection or non-infectious inflammation) was an independent determinant of the serum sMR concentration on the day of antimicrobial initiation or matched ICU day (Additional file 4). More specifically, higher values were seen in patients with IFI than in those without IFI with a β-coefficient (95% confidence interval (CI)) of 0.0325 (0.0168–0.0481), standardised-β of 0.2430 and *p* < 0.0001 (Additional file 5). Also among infected patients, presence of an IFI remained an independent determinant of serum sMR concentration with a β-coefficient (95%CI) of 0.0235 (0.0044–0.0425), standardised-β of 0.1893 and *p* = 0.02 (Additional file 6). Other factors independently associated with a rise in serum sMR concentration were APACHE-II score and the presence of sepsis upon admission. Emergency admission and Late-PN were independently associated with lower serum sMR levels (Additional file 6).

## Discussion

The MR is a marker of macrophage activation that is released from the human cell surface and shed into the circulation as sMR with exposure to several stimuli, among which fungal cell walls components [9–11]. In this study, we investigated whether serum sMR concentration is a clinically valuable diagnostic tool for IFI in ICU patients. We found that sMR concentrations are higher in patients with IFI as compared with patients suffering from a bacterial infection or non-infectious inflammation. The higher concentrations were seen as from 5 days before the IFI was diagnosed by the treating physician. This early rise in sMR concentration may be explained by an association between macrophage activation and the risk to develop IFI and/or the presence of a subclinical infection before clinical suspicion arises. The latter appears likely as classical methods used to confirm diagnosis and initiate treatment are time-demanding and are known to delay treatment initiation [4].

However, also severely critically ill patients who did not suffer from IFI showed elevated serum sMR concentrations, which suggest that sMR shedding from macrophages is rather non-specific. Consequently, the discriminative power appeared to be weak due to the lack of specificity given that patients suffering from a bacterial infection revealed increased serum sMR concentrations. Hence, the use of serum sMR concentration cannot be recommended to diagnose IFI, as its discriminative properties were poor among the studied ICU patients.

Indeed, increased serum sMR concentrations have been documented in patients suffering from a variety of diseases such as liver disease, hyper-inflammation, pneumococcal bacteraemia, malignancies and sepsis [12–18]. We evaluated the independent association between these characteristics and sMR concentration with the use of multiple linear regression analysis.

Severity of illness, as reflected by the APACHE-II score, was independently associated with sMR concentration, with increasing concentrations for higher disease severity. This is expected, as higher sMR concentrations have been shown for ICU patients as compared with healthy controls and with patients admitted to a medical ward [18]. Indeed, severe illness causes inflammation and tissue damage, activates macrophages and as such induces non-specific shedding of the receptor. Sepsis upon admission was also significantly associated with higher serum sMR concentration, confirming findings from previous studies [13–15].

Remarkably, withholding supplemental PN until beyond 1 week in ICU appeared to have a significant impact on serum sMR, with lower concentrations when PN was postponed. This may suggest a reduced macrophage activation or a reduced fungal/bacterial load when omitting PN. Previous research has shown an association between the sMR levels and mortality, with higher mortality when sMR increased [14, 15]. The lower sMR concentrations could be an important factor in the improved outcome that was observed in the Late-PN patients in the EPaNIC randomised controlled trial [19]. As far as we know, there are no data on the impact of PN on macrophage activity in humans, so further research is necessary to investigate this possibility.

Another factor significantly associated with a lower serum sMR concentration was emergency admission. However, this finding is difficult to interpret given that more than 95% of the studied patients were admitted non-electively.

In invasive pneumococcal disease, serum sMR concentrations have been shown to be lower in patients older than 75 years as compared to the younger patients [14]. Our studied population was younger, with a median age of 65 years, and we did not find an association between age and serum sMR concentration. Also, history of malignancy in the studied ICU patient cohort was not associated with sMR concentration, unlike in previous studies that reported elevated serum sMR in patients with gastric cancer and multiple myeloma [16, 17]. However, serum sMR concentrations documented in these patients were much lower than serum sMR concentrations that we documented in the cohort of ICU patients suggesting that critical illness is a stronger inducer of MR shedding than cancer.

Our study has some limitations. First, the groups were selected by propensity score matching to avoid confounding. Indeed, patients acquiring an IFI are severely ill and must be compared to equally sick patients. Although groups were well matched for several a priori defined risk factors, other unknown characteristics may still have differed. Second, patients who did not acquire an infection during ICU stay were matched to the infected patients for a similar ICU day. This was most appropriate because duration of illness may otherwise be a confounder. However, we cannot exclude that serum sMR may have declined in patients with non-infectious inflammation due to recovery. Third, the group of patients with IFI consisted of patients with proven and probable IFI. When comparing sMR concentrations in patients with proven invasive aspergillosis, probable invasive aspergillosis, candidaemia and other invasive candida diseases, sMR appeared numerically higher in patients with proven invasive aspergillosis and with candidaemia as compared to other patient groups. However, no statistical analyses could be performed given the too small numbers for this comparison. The lower serum sMR concentrations in patients with probable as compared with proven invasive aspergillosis and in patients with invasive candidiasis as compared with candidaemia could have been a consequence of less severe infections. However, misdiagnosis could also play a role given that available diagnostic tools are lacking sensitivity and specificity [4]. Theoretically, patients who did not survive ICU stay may have had an unrecognised IFI. Routine post-mortem examinations were not available to exclude this possibility. Fourth, this is a secondary analysis of the EPaNIC RCT that included many patients from a surgical ICU. However, the sickest patients were selected by propensity score matching and the final cohort consisted of a mixed ICU population with 32.2% of all patients admitted to a medical ICU.

Our study is the first clinical study to investigate serum sMR concentrations in patients with IFI. Although cell culture experiments have shown an increased shedding of MR in different cell types after exposure to fungi, studies investigating this aspect in patients were lacking. We found that the serum sMR concentration increases significantly in critically ill patients with IFI as compared with those without IFI, and this already 5 days before clinical diagnosis. We showed a significant impact of sepsis upon admission and of severity of illness on serum sMR, and a significant lowering of serum sMR with postponing administration of PN.

## Conclusions

Serum sMR concentrations are higher in ICU patients who suffer from an IFI than in those with a bacterial infection or with non-infectious inflammation. This clinical finding confirms earlier experimental evidence for a rise in MR shedding in response to fungal exposure. However, the power of serum sMR concentrations to discriminate IFI from other infections or other causes of inflammation was insufficient to advise it as a clinically reliable diagnostic tool.

## Additional files

Additional file 1: Overview of the sMR concentrations in the different sites of bacterial infections. sMR: soluble mannose receptor, IQR: interquartile range (DOCX 15 kb)

Additional file 2: sMR concentrations in the three studied patient groups from one until 5 days preceding the matching day. Serum sMR concentrations in patients with non-infectious inflammation (green), bacterial infections (bleu) and invasive fungal infections (red). Yellow-shaded bars represent the interquartile ranges of the sMR concentration of 59 healthy controls. Double-sided *p* values were calculated with Student *t* test with double square root transformed data. (DOCX 338 kb)

Additional file 3: Performance of serum sMR for the diagnosis of IFI in critically ill patients. 3a: Test characteristics of sMR for the diagnosis of IFI in all patients on the day of antimicrobial initiation for patients with a new infection and matched ICU day for patients without a new infection. aROC 0.65 (95% CI 0.59–0.70). 3b: Test characteristics of sMR for the diagnosis of IFI in all patients on the day before the day of antimicrobial initiation for patients with a new infection and matched ICU day for patients without a new infection. aROC 0.67 (95% CI 0.62–0.72). 3c: Test characteristics of sMR for the diagnosis of IFI in all patients 2 days before the day of antimicrobial initiation for patients with a new infection and matched ICU day for patients without a new infection. aROC 0.70 (95% CI 0.65–0.75). 3d: Test characteristics of sMR for the diagnosis of IFI in all patients 3 days before the day of antimicrobial initiation for patients with a new infection and matched ICU day for patients without a new infection. aROC 0.72 (95% CI 0.67–0.77). 3e: Test characteristics of sMR for the diagnosis of IFI in all patients 4 days before the day of antimicrobial initiation for patients with a new infection and matched ICU day for patients without a new infection. aROC 0.73 (95% CI 0.68–0.78). 3f: Test characteristics of sMR for the diagnosis of IFI in all patients 5 days before the day of antimicrobial initiation for patients with a new infection and matched ICU day for patients without a new infection. aROC 0.70 (95% CI 0.65–0.75). Performance is shown for sMR concentrations for the day of antimicrobial initiation and for each of the five preceding days. CI: confidence interval, PPV: positive predictive value, NPV: negative predictive value, LR +: positive likelihood ratio, LR -: negative likelihood ratio. (DOCX 29 kb)

Additional file 4: Multivariable linear regression analysis to identify characteristics independently associated with sMR concentration. Statistical analyses were performed after double square root transformation of the sMR concentrations to obtain a normal distribution. CI: confidence interval, IFI: invasive fungal infection, BMI: body mass index, APACHE-II: acute physiology and chronic health evaluation II, MV: mechanical ventilation, PN: parenteral nutrition. (DOCX 15 kb)

Additional file 5: Multivariable linear regression analysis to identify baseline characteristics independently associated with the sMR concentration on the day of antimicrobial initiation or matched ICU day. The group with no IFI represents all patients with a bacterial infection or non-infectious inflammation. Statistical analyses were performed after double square root transformation of the sMR concentrations to obtain a normal distribution. CI: confidence interval, IFI: invasive fungal infection, BMI: body mass index, APACHE-II: acute physiology and chronic health evaluation II, MV: mechanical ventilation, PN: parenteral nutrition. (DOCX 16 kb)

Additional file 6: Multivariable linear regression analysis to identify baseline characteristics independently associated with the sMR concentration on the day of antimicrobial initiation or matched ICU day, excluding patients with non-infectious inflammation. Statistical analyses were performed after double square root transformation of the sMR concentrations to obtain a normal distribution. CI: confidence interval, IFI: invasive fungal infection, BMI: body mass index, APACHE-II: acute physiology and chronic health evaluation II, MV: mechanical ventilation, PN: parenteral nutrition. (DOCX 16 kb)

## Data Availability

Greet De Vlieger had full access to all the data in the study. All data generated or analysed during this study are included in this article.

## Abbreviations

ANOVA: Analysis of variance
APACHE-II: Acute physiology and chronic health evaluation II
aROC: Area under the receiver-operating-curve
BMI: Body mass index
CI: Confidence interval
ELISA: Enzyme-linked immunosorbent assay
EORTC/MSG: European Organisation for Research and Treatment of Cancer/Mycosis Study Group
ESCMID: European Society of Clinical Microbiology and Infectious Diseases
ICU: Intensive care unit
IFI: Invasive fungal infections
IQR: Interquartile range
MR: Mannose receptor
PN: Parenteral nutrition
SD: Standard deviation
sMR: Soluble mannose receptor
