# Supplementary material for: The soluble mannose receptor (sMR/sCD206) in critically ill patients with invasive fungal infections, bacterial infections or non-infectious inflammation: a secondary analysis of the EPaNIC RCT
Source: Crit Care. 2019 Aug 2;23:270. doi: 10.1186/s13054-019-2549-8 (PMC6679534; doi:10.1186/s13054-019-2549-8)
Supplement: Supplementary file 1 — Overview of the sMR concentrations in the different sites of bacterial infections. sMR: soluble mannose receptor, IQR: interquartile range (DOCX 15 kb) [file 13054_2019_2549_MOESM1_ESM.docx]

**Additional file 1:** Overview of the sMR concentrations in the different sites of bacterial infections.

|  | Number of patients | sMR (mg/L)  (median and IQR) |
| --- | --- | --- |
| Blood stream infection | 18 | 0.82 (0.49-1.50) |
| Respiratory infection | 43 | 0.83 (0.47-1.27) |
| Abdominal infection | 4 | 0.71 (0.34-1.24) |
| Soft tissue and urinary tract infection | 7 | 0.81 (0.73-1.30) |
| Unknown | 8 | 1.07 (0.77-1.45) |

sMR: soluble mannose receptor, IQR: interquartile range
