# Supplementary material for: The soluble mannose receptor (sMR/sCD206) in critically ill patients with invasive fungal infections, bacterial infections or non-infectious inflammation: a secondary analysis of the EPaNIC RCT
Source: Crit Care. 2019 Aug 2;23:270. doi: 10.1186/s13054-019-2549-8 (PMC6679534; doi:10.1186/s13054-019-2549-8)
Supplement: Supplementary file 2 — sMR concentrations in the three studied patient groups from one until 5 days preceding the matching day. Serum sMR concentrations in patients with non-infectious inflammation (green), bacterial infections (bleu) and invasive fungal infections (red). Yellow-shaded bars represent the interquartile ranges of the sMR concentration of 59 healthy controls. Double-sided p values were calculated with Student t test with double square root transformed data. (DOCX 338 kb) [file 13054_2019_2549_MOESM2_ESM.docx]

**Additional file 2**: sMR concentrations in the three studied patient groups from one until five days preceding the matching day.


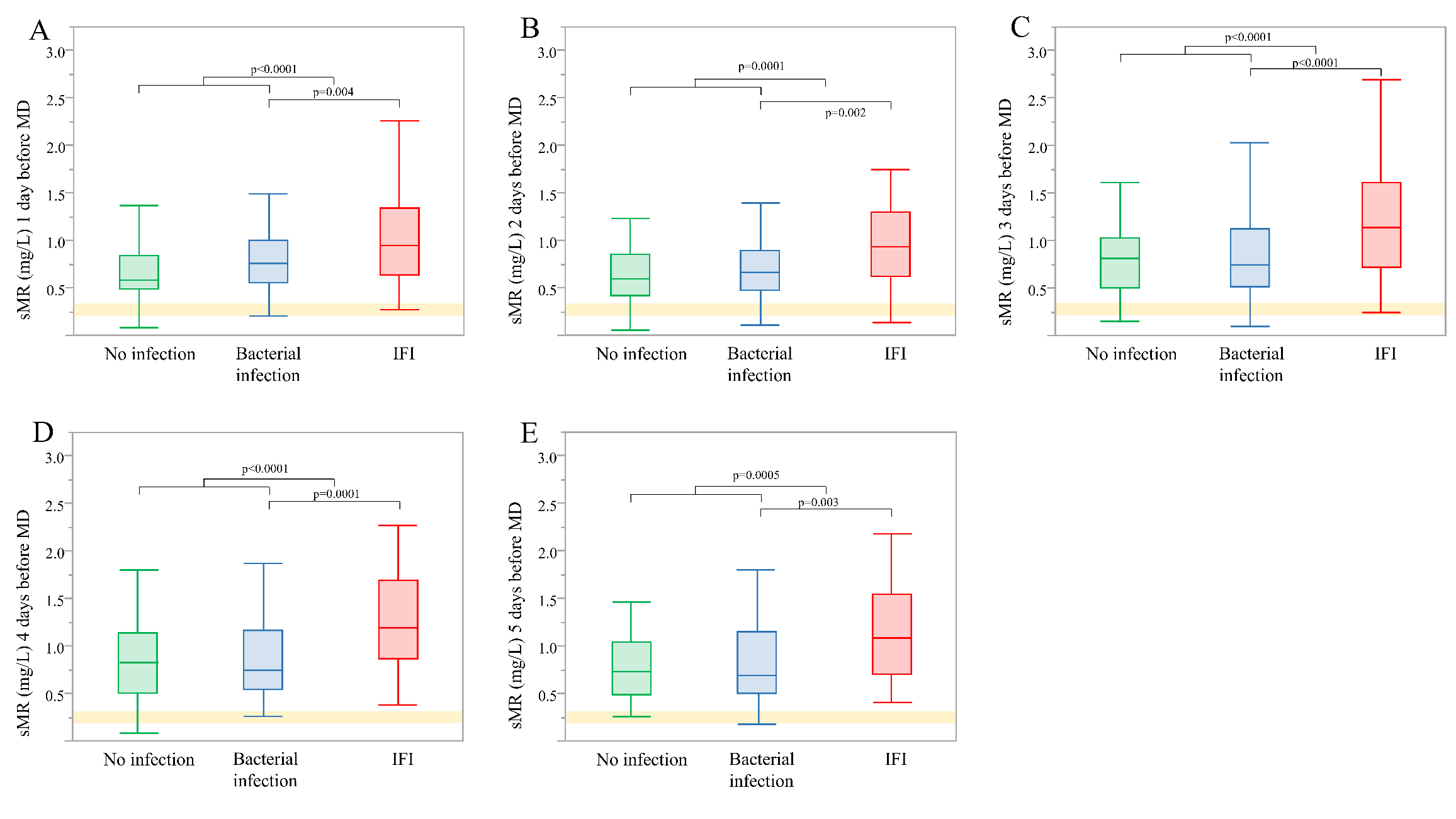


Serum sMR concentrations in patients with non-infectious inflammation (green), bacterial infections (bleu) and invasive fungal infections (red). Yellow-shaded bars represent the interquartile ranges of the sMR concentration of 59 healthy controls. Double-sided P values were calculated with student t test with double square root transformed data
