# Supplementary material for: The soluble mannose receptor (sMR/sCD206) in critically ill patients with invasive fungal infections, bacterial infections or non-infectious inflammation: a secondary analysis of the EPaNIC RCT
Source: Crit Care. 2019 Aug 2;23:270. doi: 10.1186/s13054-019-2549-8 (PMC6679534; doi:10.1186/s13054-019-2549-8)
Supplement: Supplementary file 3 — Performance of serum sMR for the diagnosis of IFI in critically ill patients. 3a: Test characteristics of sMR for the diagnosis of IFI in all patients on the day of antimicrobial initiation for patients with a new infection and matched ICU day for patients without a new infection. aROC 0.65 (95% CI 0.59–0.70). 3b: Test characteristics of sMR for the diagnosis of IFI in all patients on the day before the day of antimicrobial initiation for patients with a new infection and matched ICU day for patients without a new infection. aROC 0.67 (95% CI 0.62–0.72). 3c: Test characteristics of sMR for the diagnosis of IFI in all patients 2 days before the day of antimicrobial initiation for patients with a new infection and matched ICU day for patients without a new infection. aROC 0.70 (95% CI 0.65–0.75). 3d: Test characteristics of sMR for the diagnosis of IFI in all patients 3 days before the day of antimicrobial initiation for patients with a new infection and matched ICU day for patients without a new infection. aROC 0.72 (95% CI 0.67–0.77). 3e: Test characteristics of sMR for the diagnosis of IFI in all patients 4 days before the day of antimicrobial initiation for patients with a new infection and matched ICU day for patients without a new infection. aROC 0.73 (95% CI 0.68–0.78). 3f: Test characteristics of sMR for the diagnosis of IFI in all patients 5 days before the day of antimicrobial initiation for patients with a new infection and matched ICU day for patients without a new infection. aROC 0.70 (95% CI 0.65–0.75). Performance is shown for sMR concentrations for the day of antimicrobial initiation and for each of the five preceding days. CI: confidence interval, PPV: positive predictive value, NPV: negative predictive value, LR +: positive likelihood ratio, LR -: negative likelihood ratio. (DOCX 29 kb) [file 13054_2019_2549_MOESM3_ESM.docx]

**Additional file 3:** Performance of serum sMR for the diagnosis of IFI in critically ill patients.

**3a:** Test characteristics of sMR for the diagnosis of IFI in all patients on the day of antimicrobial initiation for patients with a new infection and matched ICU day for patients without a new infection. aROC 0.65 (95% CI 0.59-0.70).

| Cutoff  (mg/L) | Sensitivity (%)  (95% CI) | Specificity (%)  (95% CI) | PPV (%)  (95% CI) | NPV (%)  (95% CI) | LR +  (95% CI) | LR –  (95% CI) |
| --- | --- | --- | --- | --- | --- | --- |
| 2.50 | 1.2 (0.2-6.6) | 100 (97.6-100) | 100 (20.7-100) | 66.0 (59.7-71.7) | infinite | 0.99 (0.96-1.01) |
| 2.25 | 2.4 (0.3-8.5) | 98.7 (95.5-99.9) | 50.0 (12.6-87.5) | 66.0 (65.0-66.8) | 1.91 (0.27-13.35) | 0.99 (0.95-1.03) |
| 2.00 | 2.4 (0.7-8.5) | 97.5 (93.6-99.0) | 33.3 (9.7-70.0) | 65.7 (59.4-71.5) | 0.96 (0.18-5.12) | 1.00 (0.96-1.04) |
| 1.75 | 11.0 (5,9-19.6) | 97.5 (93.6-99.0) | 69.2 (42.4-87.3) | 67.7 (61.4-73.5) | 4.31 (1.37-13.57) | 0.91 (0.84-0.99) |
| 1.50 | 19.5 (12.4-29.4) | 95.5 (91.1-97.8) | 69.6 (49.1-84.4) | 69.4 (63.0-75.2) | 4.38 (1.88-10.21) | 0.84 (0.75-0.94) |
| 1.25 | 25.6 (17.4-36.0) | 89.8 (84.1-93.6) | 56.8 (40.9-71.3) | 69.8 (63.2-75.7) | 2.51 (1.39-4.55) | 0.83 (0.72-0.95) |
| 1.00 | 42.7 (32.5-53.5) | 79.0 (72.0-84.6) | 51.5 (39.8-62.9) | 72.5 (65.4-78.7) | 2.03 (1.37-3.01) | 0.73 (0.59-0.89) |
| 0.75 | 63.4 (52.6-73.0) | 56.1 (48.2-63.6) | 43.0 (34.5-51.9) | 74.6 (66.0-81.6) | 1.44 (1.13-1.84) | 0.65 (0.48-0.90) |
| 0.50 | 89.0 (80.4-94.1) | 28.0 (21.6-35.5) | 39.2 (32.5-46.4) | 83.0 (70.8-90.8) | 1.24 (1.09-1.40) | 0.39 (0.20-0.76) |
| 0.25 | 100 (95.5-100) | 3.8 (1.8-8.1) | 35.2 (29.3-41.5) | 100 (61.0-100) | 1.04 (1.01-1.07) | 0 (0-0) |

**3b:** Test characteristics of sMR for the diagnosis of IFI in all patients on the day before the day of antimicrobial initiation for patients with a new infection and matched ICU day for patients without a new infection. aROC 0.67 (95% CI 0.62-0.72).

| Cutoff  (mg/L) | Sensitivity (%)  (95% CI) | Specificity (%)  (95% CI) | PPV (%)  (95% CI) | NPV (%)  (95% CI) | LR +  (95% CI) | LR –  (95% CI) |
| --- | --- | --- | --- | --- | --- | --- |
| 2.50 | 1.5 (0.04-8.2) | 99.3 (96.3-100) | 50.0 (6.0-94.0) | 69.3 (68.6-70.0) | 2.24 (0.14-35.3) | 0.99 (0.96-1.02) |
| 2.25 | 4.6 (1.0-12.7) | 98.7 (95.2-99.8) | 60.0 (20.4-89.8) | 69.9 (68.7-71.0) | 3.36 (0.58-19.66) | 0.97 (0.91-1.02) |
| 2.00 | 7.6 (2.5-16.8) | 98.0 (94.2-99.6) | 62.5 (29.1-87.1) | 70.4 (68.9-71.9) | 3.74 (0.92-15.18) | 0.94 (0.88-1.01) |
| 1.75 | 12.1 (5.4-22.5) | 98.0 (94.2-99.6) | 72.7 (42.2-90.7) | 71.4 (69.5-73.3) | 5.98 (1.64-21.83) | 0.90 (0.82-0.98) |
| 1.50 | 16.7 (8.6-27.9) | 96.0 (91.4-98.5) | 64.7 (41.5-82.6) | 72.1 (69.8-74.3) | 4.11 (1.59-10.65) | 0.87 (0.78-0.97) |
| 1.25 | 30.3 (19.6-42.9) | 91.9 (86.3-95.7) | 62.5 (46.4-76.2) | 74.7 (71.5-77.7) | 3.74 (1.94-7.19) | 0.76 (0.64-0.90) |
| 1.00 | 45.5 (33.1-58.2) | 81.1 (73.8-87.1) | 51.7 (41.2-62.1) | 76.9 (72.5-80.8) | 2.40 (1.57-3.68) | 0.67 (0.53-0.85) |
| 0.75 | 60.6 (47.8-72.4) | 57.4 (49.1-65.5) | 38.8 (32.7-45.4) | 76.6 (70.2-82.0) | 1.42 (1.09-1.86) | 0.69 (0.49-0.95) |
| 0.50 | 87.9 (77.5-94.6) | 26.4 (19.5-34.2) | 34.7 (31.8-37.8) | 83.0 (70.7-90.8) | 1.19 (1.05-1.36) | 0.60 (0.23-0.93) |
| 0.25 | 100 (94.5-100) | 2.7 (1.1-6.7) | 31.4 (25.5-38.0) | 100 (51.0-100) | 1.03 (1.00-1.06) | 0 (0-0) |

**3c:** Test characteristics of sMR for the diagnosis of IFI in all patients two days before the day of antimicrobial initiation for patients with a new infection and matched ICU day for patients without a new infection. aROC 0.70 (95% CI 0.65-0.75).

| Cutoff  (mg/L) | Sensitivity (%)  (95% CI) | Specificity (%)  (95% CI) | PPV (%)  (95% CI) | NPV (%)  (95% CI) | LR +  (95% CI) | LR –  (95% CI) |
| --- | --- | --- | --- | --- | --- | --- |
| 2.50 | 3.5 (0.4-11.9) | 99.3 (95.9-100.0) | 66.7 (15.6-95.6) | 70.4 (69.3-71.4) | 4.62 (0.43-49.96) | 0.97 (0.92-1.02) |
| 2.25 | 3.5 (0.4-11.9) | 98.5 (94.7-99.8) | 50.0 (12.6-87.4) | 70.2 (69.1-71.3) | 2.32 (0.33-16.01) | 0.98 (0.93-1.03) |
| 2.00 | 3.5 (0.4-11.9) | 97.8 (93.6-99.5) | 40.0 (10.3-79.5) | 70.1 (68.9-71.2) | 1.54 (0.26-8.97) | 0.99 (0.93-1.04) |
| 1.75 | 6.9 (1.9-16.7) | 97.8 (93.6-99.5) | 57.1 (23.6-85.2) | 70.8 (69.3-72.3) | 3.08 (0.71-13.3) | 0.95 (0.88-1.03) |
| 1.50 | 19.0 (9.9-31.4) | 97.0 (92.5-99.2) | 73.3 (47.7-89.2) | 73.5 (70.9-75.9) | 6.35 (2.11-19.13) | 0.84 (0.73-0.95) |
| 1.25 | 32.8 (21.0-46.3) | 91.8 (85.8-95.8) | 63.3 (46.8-77.3) | 75.9 (72.4-79.2) | 3.99 (2.03-7.84) | 0.73 (0.61-0.88) |
| 1.00 | 50.0 (36.6-63.4) | 79.9 (72.1-86.3) | 51.8 (41.3-62.1) | 78.7 (73.8-82.9) | 2.48 (1.62-3.79) | 0.63 (0.48-0.82) |
| 0.75 | 72.4 (59.1-83.3) | 60.5 (51.6-68.8) | 44.2 (37.9-50.8) | 83.5 (76.6-88.7) | 1.83 (1.41-2.38) | 0.46 (0.29-0.71) |
| 0.50 | 87.9 (76.7-95.0) | 27.6 (20.2-36.0) | 34.5 (31.3-37.7) | 84.1 (71.5-91.8) | 1.21 (1.05-1.40) | 0.44 (0.21-0.92) |
| 0.25 | 96.6 (88.1-99.6) | 3.0 (0.8-7.5) | 30.1 (28.9-31.3) | 66.7 (27.4-91.4) | 1.0 (0.94-1.05) | 1.16 (0.22-6.13) |

**3d:** Test characteristics of sMR for the diagnosis of IFI in all patients three days before the day of antimicrobial initiation for patients with a new infection and matched ICU day for patients without a new infection. aROC 0.72 (95% CI 0.67-0.77).

| Cutoff  (mg/L) | Sensitivity (%)  (95% CI) | Specificity (%)  (95% CI) | PPV (%)  (95% CI) | NPV (%)  (95% CI) | LR +  (95% CI) | LR –  (95% CI) |
| --- | --- | --- | --- | --- | --- | --- |
| 2.50 | 0 (0-7.1) | 99.1 (95.3-100) | 0 (0-0) | 69.7 (69.3-70.1) | 0 | 1.01 (0.99-1.03) |
| 2.25 | 4.0 (0.5-13.7) | 98.3 (93.9-99.8) | 50.0 (12.7-87.3) | 70.4 (69.1-71.6) | 2.32 (0.34-16.01) | 0.98 (0.92-1.04) |
| 2.00 | 6.0 (1.25-16.6) | 98.3 (93.9-99.8) | 60.0 (20.5-89.7) | 70.8 (69.3-72.3) | 3.48 (0.60-20.19) | 0.96 (0.89-1.03) |
| 1.75 | 8.0 (2.2-19.2) | 97.4 (92.6-99.5) | 57.1 (23.7-85.2) | 71.1 (69.3-72.8) | 3.09 (0.72-13.32) | 0.94 (0.87-1.03) |
| 1.50 | 22.0 (11.5-36.0) | 94.0 (88.0-97.5) | 61.1 (39.3-79.2) | 73.7 (70.6-76.5) | 3.65 (1.50-8.86) | 0.83 (0.71-0.97) |
| 1.25 | 36.0 (22.9-50.8) | 89.7 (82.6-94.5) | 60.0 (43.9-74.2) | 76.5 (72.4-80.2) | 3.48 (1.8-6.67) | 0.71 (0.57-0.89) |
| 1.00 | 56.0 (41.3-70.0) | 76.7 (68.0-84.1) | 50.9 (40.7-61.0) | 80.2 (84.4-84.9) | 2.41 (1.59-3.63) | 0.57 (0.41-0.80) |
| 0.75 | 74.0 (59.7-85.4) | 52.6 (43.1-61.9) | 40.2 (34.3-46.4) | 82.4 (74.0-88.5) | 1.56 (1.21-2.01) | 0.49 (0.30-0.81) |
| 0.50 | 90.0 (78.2-96.7) | 31.0 (22.8-40.3) | 36.0 (32.6-39.6) | 87.8 (75.0-94.5) | 1.30 (1.12-1.52) | 0.32 (0.13-0.77) |
| 0.25 | 98.0 (89.4-100.0) | 4.3 (1.4-9.8) | 30.6 (29.5-31.8) | 83.3 (37.5-97.7) | 1.02 (0.97-1.08) | 0.46 (0.06-3.87) |

| Cutoff  (mg/L) | Sensitivity (%)  (95% CI) | Specificity (%)  (95% CI) | PPV (%)  (95% CI) | NPV (%)  (95% CI) | LR +  (95% CI) | LR –  (95% CI) |
| --- | --- | --- | --- | --- | --- | --- |
| 2.50 | 2.4 (0.06-12.9) | 100 (96.5-100) | 100 (100-100) | 71.8 (70.8-72.8) | 0 | 0.98 (0.93-1.02) |
| 2.25 | 2.4 (0.06-12.9) | 100 (96.5-100) | 100 (100-100) | 71.8 (70.8-72.8) | 0 | 0.98 (0.93-1.02) |
| 2.00 | 2.4 (0.06-12.9) | 98.0 (93.1-99.8) | 33.3 (4.5-84.3) | 71.4 (70.3-72.6) | 1.24 (0.12-13.35) | 1.00 (0.94-1.05) |
| 1.75 | 7.3 (1.5-19.9) | 97.1 (91.6-99.4) | 50.0 (17.4-82.6) | 72.3 (70.4-74.1) | 2.49 (0.52-11.82) | 0.95 (0.87-1.05) |
| 1.50 | 14.6 (5.6-29.2) | 95.1 (88.9-98.4) | 54.6 (27.9-78.8) | 73.5 (70.8-76.0) | 2.99 (0.96-9.24) | 0.90 (0.78-1.03) |
| 1.25 | 34.2 (20.1-50.6) | 92.2 (85.1-96.6) | 63.6 (44.3-79.4) | 77.7 (73.5-81.4) | 4.35 (1.98-9.59) | 0.71 (0.57-0.90) |
| 1.00 | 51.2 (35.1-67.1) | 79.4 (70.3-86.8) | 50.0 (38.1-61.9) | 80.2 (74.5-84.9) | 2.49 (1.53-4.04) | 0.61 (0.44-0.85) |
| 0.75 | 70.7 (54.5-83.9) | 57.8 (47.7-67.6) | 40.3 (33.3-47.7) | 83.1 (74.0-89.1) | 1.68 (1.24-2.27) | 0.51 (0.31-0.84) |
| 0.50 | 92.7 (80.1-98.5) | 32.4 (23.4-42.3) | 35.5 (32.0-39.2) | 91.7 (78.1-97.1) | 1.37 (1.17-1.61) | 0.23 (0.07-0.70) |
| 0.25 | 100 (91.4-100) | 5.9 (2.2-12.4) | 29.9 (28.9-31.0) | 100 (100-100) | 1.06 (1.01-1.12) | 0 |

**3e:** Test characteristics of sMR for the diagnosis of IFI in all patients four days before the day of antimicrobial initiation for patients with a new infection and matched ICU day for patients without a new infection. aROC 0.73 (95% CI 0.68-0.78).

**3f:** Test characteristics of sMR for the diagnosis of IFI in all patients five days before the day of antimicrobial initiation for patients with a new infection and matched ICU day for patients without a new infection. aROC 0.70 (95% CI 0.65-0.75).

| Cutoff  (mg/L) | Sensitivity (%)  (95% CI) | Specificity (%)  (95% CI) | PPV (%)  (95% CI) | NPV (%)  (95% CI) | LR +  (95% CI) | LR –  (95% CI) |
| --- | --- | --- | --- | --- | --- | --- |
| 2.50 | 0 (0-9.3) | 97.7 (91.9-99.7) | 0 | 69.1 (68.4-69.8) | 0 | 1.02 (0.99-1.06) |
| 2.25 | 0 (0-9.3) | 96.6 (90.3-99.3) | 0 | 68.8 (68.0-69.7) | 0 | 1.04 (1.00-1.08) |
| 2.00 | 2.6 (0.07-13.8) | 96.6 (90.3-99.3) | 25.0 (3.5-75.6) | 69.4 (68.0-70.8) | 0.76 (0.08-7.10) | 1.01 (0.94-1.08) |
| 1.75 | 7.9 (1.7-21.4) | 96.6 (90.3-99.3) | 50.0 (17.5-82.6) | 70.6 (68.4-72.6) | 2.29 (0.48-10.83) | 0.95 (0.86-1.06) |
| 1.50 | 15.8 (6.0-32.3) | 93.1 (95.6-97.4) | 50.0 (25.6-74.4) | 71.7 (68.6-74.6) | 2.29 (0.79-6.64) | 0.90 (0.78-1.05) |
| 1.25 | 36.8 (21.8-54.0) | 89.7 (81.3-95.2) | 60.9 (42.5-76.6) | 76.5 (71.6-80.7) | 3.56 (1.69-7.51) | 0.70 (0.55-0.91) |
| 1.00 | 50.0 (33.4-66.6) | 74.7 (64.3-83.4) | 46.3 (34.8-58.3) | 77.4 (70.9-82.8) | 1.98 (1.22-3.20) | 0.67 (0.48-0.94) |
| 0.75 | 68.4 (51.4-82.5) | 63.2 (52.2-73.3) | 44.8 (36.4-53.6) | 82.1 (73.7-88.3) | 1.86 (1.31-2.64) | 0.50 (0.30-0.82) |
| 0.50 | 86.8 (71.9-95.6) | 35.6 (25.7-46.6) | 37.1 (32.6-41.8) | 86.1 (72.3-93.6) | 1.35 (1.11-1.65) | 0.37 (0.16-0.88) |
| 0.25 | 100 (90.8-100.0) | 3.5 (0.7-9.8) | 31.2 (30.3-32.0) | 100 (100-100) | 1.04 (1.00-1.08) | 0 |

Performance is shown for sMR concentrations for the day of antimicrobial initiation and for each of the five preceding days. CI: confidence interval, PPV: positive predictive value, NPV: negative predictive value, LR +: positive likelihood ratio, LR -: negative likelihood ratio
