# Supplementary material for: The soluble mannose receptor (sMR/sCD206) in critically ill patients with invasive fungal infections, bacterial infections or non-infectious inflammation: a secondary analysis of the EPaNIC RCT
Source: Crit Care. 2019 Aug 2;23:270. doi: 10.1186/s13054-019-2549-8 (PMC6679534; doi:10.1186/s13054-019-2549-8)
Supplement: Supplementary file 4 — Multivariable linear regression analysis to identify characteristics independently associated with sMR concentration. Statistical analyses were performed after double square root transformation of the sMR concentrations to obtain a normal distribution. CI: confidence interval, IFI: invasive fungal infection, BMI: body mass index, APACHE-II: acute physiology and chronic health evaluation II, MV: mechanical ventilation, PN: parenteral nutrition. (DOCX 15 kb) [file 13054_2019_2549_MOESM4_ESM.docx]

|  | **β-coefficient (95% CI)** | **Standardised β** | **P-value** |
| --- | --- | --- | --- |
| Patient group |  |  |  |
| *IFI vs no infection* | *0.0431 (0.0225 — 0.0638)* | *0.2775* | *<0.0001* |
| Bacterial vs no infection | 0.0002 (-0.0214 — 0.0217) | 0.0011 | 0.99 |
| Age | -0.0009 (-0.0019 — 0.0002) | -0.1031 | 0.10 |
| BMI | -0.0005 (-0.0031 — 0.0021) | -0.0230 | 0.71 |
| Malignancy | -0.0043(-0.0225 — 0.0139) | -0.0298 | 0.64 |
| Cirrhosis Child-Pugh B or C | 0.0092 (-0.0145 — 0.0329) | 0.0474 | 0.45 |
| Diabetes mellitus | -0.0069 (-0.0288 — 0.0150) | -0.0389 | 0.54 |
| *APACHE-II* | *0.0059 (0.0035 — 0.0085)* | *0.3611* | *<0.0001* |
| *Sepsis upon admission* | *0.0282 (0.0072 — 0.0492)* | *0.1709* | *0.009* |
| *Emergency admission* | *-0.0515 (-0.0928 — -0.0103)* | *-0.1704* | *0.01* |
| Diagnostic group |  |  |  |
| Cardiac surgery vs medical | 0.0019 (-0.0490 — 0.0529) | 0.0086 | 0.94 |
| Complicated surgery vs medical | -0.0023 (-0.050 — 0.0045) | -0.1621 | 0.12 |
| Trauma/burns vs medical | 0.0286 (-0.0184 — 0.0756) | 0.1284 | 0.23 |
| Steroids upon admission | -0.0045 (-0.0207 — 0.0117) | -0.0335 | 0.59 |
| MV upon admission | -0.0050 (-0.0290 — 0.0191) | -0.0295 | 0.68 |
| *Randomisation Late-PN vs Early-PN* | *-0.0167 (-0.0314 — -0.0021)* | *-0.1319* | *0.02* |

**Additional file 4:** Multivariable linear regression analysis to identify characteristics independently associated with sMR concentration.

Statistical analyses were performed after double square root transformation of the sMR concentrations to obtain a normal distribution. CI: confidence interval, IFI: invasive fungal infection, BMI: body mass index, APACHE-II: acute physiology and chronic health evaluation II, MV: mechanical ventilation, PN: parenteral nutrition.
