# Supplementary material for: The soluble mannose receptor (sMR/sCD206) in critically ill patients with invasive fungal infections, bacterial infections or non-infectious inflammation: a secondary analysis of the EPaNIC RCT
Source: Crit Care. 2019 Aug 2;23:270. doi: 10.1186/s13054-019-2549-8 (PMC6679534; doi:10.1186/s13054-019-2549-8)
Supplement: Supplementary file 5 — Multivariable linear regression analysis to identify baseline characteristics independently associated with the sMR concentration on the day of antimicrobial initiation or matched ICU day. The group with no IFI represents all patients with a bacterial infection or non-infectious inflammation. Statistical analyses were performed after double square root transformation of the sMR concentrations to obtain a normal distribution. CI: confidence interval, IFI: invasive fungal infection, BMI: body mass index, APACHE-II: acute physiology and chronic health evaluation II, MV: mechanical ventilation, PN: parenteral nutrition. (DOCX 16 kb) [file 13054_2019_2549_MOESM5_ESM.docx]

**Additional file 5:** Multivariable linear regression analysis to identify baseline characteristics independently associated with the sMR concentration on the day of antimicrobial initiation or matched ICU day. The group with no IFI represents all patients with a bacterial infection or non-infectious inflammation.

|  | **β-coefficient (95% CI)** | **Standardised β** | **P-value** |
| --- | --- | --- | --- |
| *IFI versus no IFI* | *0.0325 (0.0168 — 0.0481)* | *0.2430* | *<0.0001* |
| Age | -0.0009 (-0.0019 — 0.00009) | -0.1133 | 0.07 |
| BMI | -0.0001 (-0.0027 — 0.0026) | -0.0043 | 0.95 |
| Malignancy | -0.0035 (-0.0219 — 0.0148) | -0.0245 | 0.71 |
| Cirrhosis child pugh B or C | 0.0115 (-0.0124 — 0.0353) | 0.0590 | 0.35 |
| Diabetes mellitus | -0.0048 (-0.0268 — 0.0173) | -0.0268 | 0.67 |
| *APACHE-II* | *0.0062 (0.0037 — 0.0087)* | *0.3725* | *<0.0001* |
| *Sepsis upon admission* | *0.0290 (0.0078 — 0.0502)* | *0.1760* | *<0.01* |
| *Emergency admission* | *-0.0492 (-0.0908 — -0.0077)* | *-0.1628* | *0.02* |
| Diagnostic group |  |  |  |
| Cardiac surgery vs medical | 0.0069 (-0.0444 — 0.0581) | 0.0305 | 0.79 |
| Complicated surgery vs medical | -0.0257 (-0.0530 — 0.0017) | -0.1833 | 0.07 |
| Trauma/burns vs medical | 0.0355 (-0.0116 — 0.0825) | 0.1591 | 0.14 |
| Steroids upon admission | -0.004 (-0.0203 — 0.0123) | -0.0301 | 0.63 |
| MV upon admission | -0.0054 (-0.0296 — 0.0189) | -0.0320 | 0.66 |
| *Randomisation Late-PN vs Early-PN* | *-0.0174 (-0.0323 — -0.0027)* | *-0.1379* | *0.02* |

Statistical analyses were performed after double square root transformation of the sMR concentrations to obtain a normal distribution. CI: confidence interval, IFI: invasive fungal infection, BMI: body mass index, APACHE-II: acute physiology and chronic health evaluation II, MV: mechanical ventilation, PN: parenteral nutrition.
