# Supplementary material for: The soluble mannose receptor (sMR/sCD206) in critically ill patients with invasive fungal infections, bacterial infections or non-infectious inflammation: a secondary analysis of the EPaNIC RCT
Source: Crit Care. 2019 Aug 2;23:270. doi: 10.1186/s13054-019-2549-8 (PMC6679534; doi:10.1186/s13054-019-2549-8)
Supplement: Supplementary file 6 — Multivariable linear regression analysis to identify baseline characteristics independently associated with the sMR concentration on the day of antimicrobial initiation or matched ICU day, excluding patients with non-infectious inflammation. Statistical analyses were performed after double square root transformation of the sMR concentrations to obtain a normal distribution. CI: confidence interval, IFI: invasive fungal infection, BMI: body mass index, APACHE-II: acute physiology and chronic health evaluation II, MV: mechanical ventilation, PN: parenteral nutrition. (DOCX 16 kb) [file 13054_2019_2549_MOESM6_ESM.docx]

**Additional file 6:** Multivariable linear regression analysis to identify baseline characteristics independently associated with the sMR concentration on the day of antimicrobial initiation or matched ICU day, excluding patients with non-infectious inflammation.

|  | **β-coefficient (95% CI)** | **Standardised β** | **P-value** |
| --- | --- | --- | --- |
| *IFI versus bacterial infection* | *0.0235 (0.0044 — 0.0425)* | *0.1893* | *0.02* |
| Age | -0.0012 (-0.0026 — 0.0001) | -0.1501 | 0.07 |
| BMI | 0.0006 (-0.0025 — 0.0037) | 0.0333 | 0.68 |
| Malignancy | -0.0116 (-0.0351 — 0.0118) | -0.0834 | 0.33 |
| Cirrhosis child pugh B or C | 0.0066 (-0.0227 — 0.0360) | 0.0360 | 0.66 |
| Diabetes mellitus | -0.0031 (-0.0297 — 0.0234) | -0.0191 | 0.64 |
| *APACHE-II* | *0.0061 (0.0027 — 0.0094)* | *0.3648* | *<0.001* |
| Sepsis upon admission | 0.0226 (-0.0035 — 0.0487) | 0.1416 | 0.09 |
| *Emergency admission* | *-0.0619 (-0.1175 — -0.0063)* | *-0.2030* | *0.03* |
| Diagnostic group |  |  |  |
| Cardiac surgery vs medical | 0.0053 (-0.0551 — 0.0658) | 0.0240 | 0.86 |
| Complicated surgery vs medical | -0.0220 (-0.0555 — 0.0114) | -0.1556 | 0.19 |
| Trauma/burns vs medical | 0.0281 (-0.0254 — 0.0817) | 0.1307 | 0.30 |
| Steroids upon admission | -0.0085 (-0.0296 — 0.0127) | -0.0647 | 0.43 |
| MV upon admission | -0.0074 (-0.0389 — 0.0241) | -0.0431 | 0.64 |
| Randomisation Late-PN vs Early-PN | -0.0175 (-0.0363 — -0.0014) | -0.1407 | 0.07 |

Statistical analyses were performed after double square root transformation of the sMR concentrations to obtain a normal distribution. CI: confidence interval, IFI: invasive fungal infection, BMI: body mass index, APACHE-II: acute physiology and chronic health evaluation II, MV: mechanical ventilation, PN: parenteral nutrition.
